# MXene-assisted organic electrochemical transistor biosensor with multiple spiral interdigitated electrodes for sensitive quantification of fPSA/tPSA

**DOI:** 10.1186/s12951-021-01121-x

**Published:** 2021-11-24

**Authors:** Yi-Cheng Zhu, Biao Cai, Quan Jiang, Yuan Zhang, Jianjun Sha, Shaowei Xie

**Affiliations:** 1 Department of Ultrasound, Pudong New Area People’s Hospital Affiliated to Shanghai University of Medicine and Health Sciences, Shanghai, 201200 China; 2 Department of Urology, Renji Hospital, Shanghai Jiao Tong University School of Medicine, Shanghai, 200127 China; 3 Department of Ultrasound, Renji Hospital, Shanghai Jiao Tong University School of Medicine, Shanghai, 200127 China

**Keywords:** Prostate specific antigen, Organic electrochemical transistor, Multiplexed spiral, Biomarker

## Abstract

**Background:**

The ratio of fPSA/tPSA in the "grey zone" of tPSA with the concentration range between 4 ng/ml and 10 ng/ml is significant for diagnosis of prostate cancer, and highly efficiency quantification of the ratio of fPSA/tPSA remain elusive mainly because of their extremely low concentration in patients' peripheral blood with high biosample complexity.

**Methods:**

We presented an interdigitated spiral-based MXene-assisted organic electrochemical transistors (isMOECTs) biosensor for highly sensitive determination of fPSA/tPSA. The combination of MXene and the interdigitated multiple spiral architecture synergistically assisted the amplification of amperometric signal of biosensor with dual functionalizations of anti-tPSA and anti-fPSA.

**Results:**

The ultrasensitivity of the biosensor was enhanced by tunable multiple spiral architecture and MXene nanomaterials; and the sensor exhibited improved detection limit of tPSA and fPSA down to 0.01 pg/ml and acceptable performance of selectivity, repeatability and stability. Moreover, the isMOECTs displayed area under the curve (AUC) value of 0.8138, confirming the potential applications of isMOECTs in clinics.

**Conclusions:**

The merits of isMOECTs biosensor demonstrated the reliability of MXene-assisted organic electrochemical transistor biosensor with multiple interdigitated spiral for ultrasensitive quantification of fPSA/tPSA, suggesting potential current and future point-of-care testing applications.

**Graphical Abstract:**

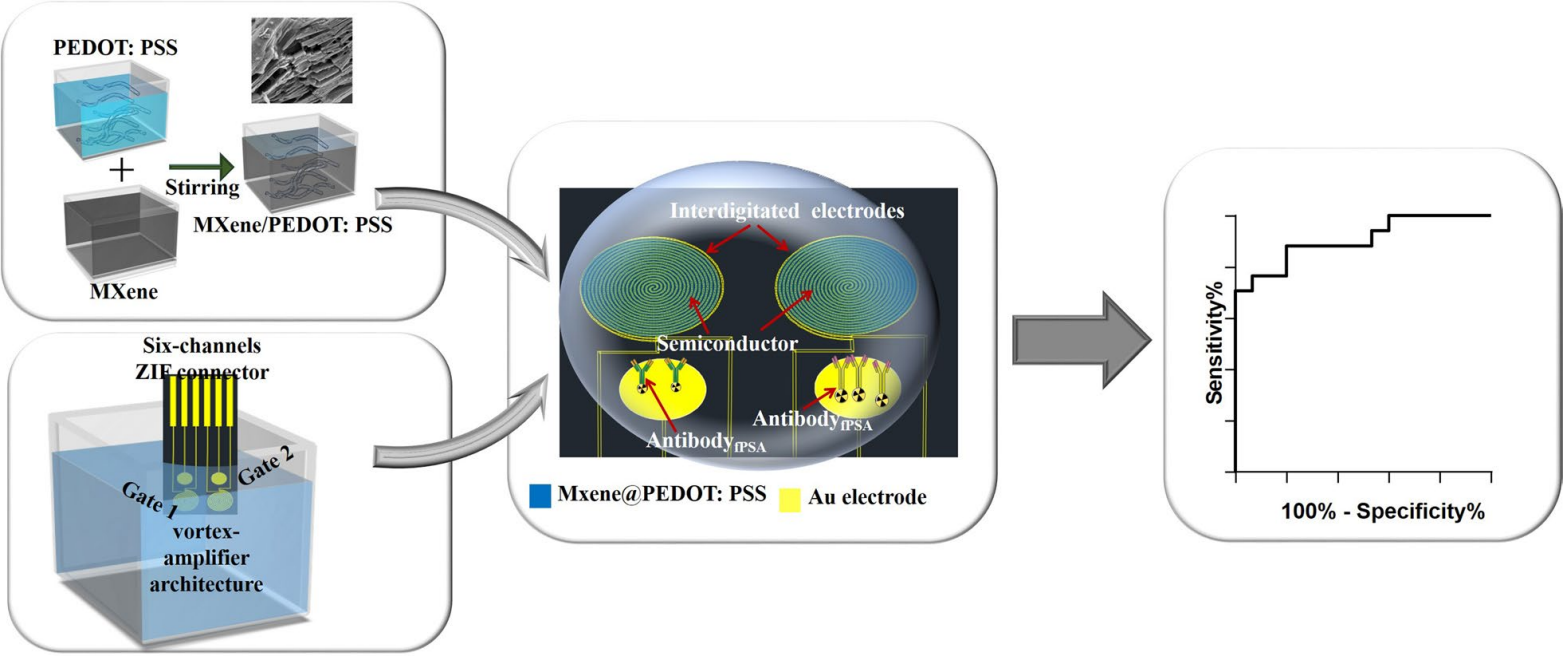

**Supplementary Information:**

The online version contains supplementary material available at 10.1186/s12951-021-01121-x.

## Introduction

Prostate cancer (PCa) ranks as the second prevalent cause of death among men. In the past twenty years, prostate specific antigen has been regarded as the most frequently used and important biomarker for screening, diagnosis, staging and prognosis of PCa [1]. The PSA is produced by the prostate to liquefy the seminal fluids, which is a glycoprotein and circulates in serum by two different molecular morphology: uncomplexed PSA-α1-antichymotrypsin complex (free PSA, fPSA) and complexed PSA (cPSA). The fPSA accouts for 10–30% of total PSA (tPSA) and other PSA lack immunological activity. For the diagnosis of PCa, the criterion for biomarker of PSA as following: the value of tPSA above 10 ng/ml is evaluated as the positive, indicating high risk of PCa; the value of tPSA below 4 ng/ml is regarded as the negative and low possibility; the value in the range of 4 ng/ml to 10 ng/ml is so-called "grey zone" and the fPSA/tPSA ratio is clinically critical for the precise diagnosis of PCa.

A variety of techniques have been developed for the determination of fPSA/tPSA including the electrochemical impedimetric immunosensors [2], bead-array fluorescence assay [3] and screen-printed electrochemical biosensor [4]. However, above-mentioned immunoassays have suffered from several shortcomings, such as limited sensitivity, sophisticated procedures/operation and hard to miniaturization. Over the past years, organic electrochemical transistors (OECTs) have attracted intensive attentions for various biosensing applications including ions, [5, 6] dopamine [7], cells [8], bacteria [9], pH [10], in virtue of its intrinsic amplification functionality, low operation voltage in aqueous environment [11–14]. The OECTs translate the small signal of ionic fluxes into an amplified electrical readout. As a typical OECTs, a wildly accepted semiconducting poly (3, 4-ethylenedioxythiophene): poly (styrenesulfonate) (PEDOT: PSS) would assist three electrodes (gate, source and drain) to transducer biochemical signal with high gain. In particularly, the gain performance could be evaluated by following equation:

1 $${G}_{\mathrm{m}}=\frac{\mathrm{W}d}{\mathrm{L}}\mu {C}^{*}({V}_{\mathrm{th}}-{V}_{\mathrm{G}})$$

G_m_, μ, C* and V_th_ are the transconductance, mobility, volumetric capacitance and threshold voltage, respectively.

From above relationship, unique structure of OECTs and the mobility decide its performance [15–17]. Previous researchers have reported interdigitated structure to improve the ratio of W/L without significantly increasing device area, which validated the feasibility of interdigitated architecture for amplification of OECTs biosensors [18, 19]. Meanwhile, electrodes with multiple spiral have been reported for biosensing applications due to the unique structure [20, 21]. However, the OECTs with interdigitated multiple spiral hasn’t reported for the biosensing of fPSA/tPSA. Distinct from 1 and 0D nanomaterials, two-dimensional (2D) nanomaterials have attracted intensive interests in the design of novel biointerfaces for bio-analytical platform its unique intercalation/delamination architecture and multi-functionality (e.g. high surface area and excellent conduction) [22–24].Transition metal carbide or nitride (MXene) has been regarded as next-generation two-dimensional (2D) nanomaterials for wide biomedical applications, including biosensing, soft electronics, supercapacitor and lithium-ion battery due to its unique characteristics [25–28]. Two-dimensional Ti_3_C_2_Tx (MXene) has been recently explored for biosensing, such as glucose [29], hemoglobin [30], gliotoxin [31] and carcinoembryonic antigen (CEA) [32]. Considering the intercalation and delamination of layered carbides and carbonitrides, MXene nanocomposites with high electrical conductivity and mobility, tunable architecture, and functional groups provide an ideal site for structural rearrangement semiconducting nanomaterials [33–36]. However, there are no reports for combination of MXene and PEDOT: PSS for synergistic determination of fPSA/tPSA.

Herein, in this paper, we proposed a dual OECTs biosensor with optimized electrode architecture and MXene-assisted semiconducting nanomaterials for the ultrasensitive quantification of fPSA/tPSA. Notably, our device demonstrated the improved detection limit of fPSA and tPSA biomarkers down to 0.01 pg/ml (S/N > 3), as well as acceptable stability, repeatability and selectivity, comparable to the latest reports. Finally, the practical efficiency of the dual OECTs biosensor were experimentally evidenced against commercial equipment and demonstrated comparable linearity and high correlation coefficient in bio-specimens quantification.

## Materials and methods

### Materials

Prostate specific antigen, cluster of differentiation 44 and 63 (CD44 and CD63, respectively), carcinoembryonic antigen (CEA), alpha-fetoprotein (AFP) were purchased from Abcam, Inc. (Cambridge, MA). Monoclonal anti-Free PSA (ab1, PSA30) and monoclonal anti-Total PSA (ab2, PSA10) were provided by Guan Xian Shenglangsai Bio-Technology Co., Ltd (Chins). The PEDOT: PSS (PH 1000) aqueous solutions was purchased from Heraeus Clevios GmbH (Germany, Leverkusen). The 3-Mercaptopropionic acid (MCA), d-sorbitol, ethylene glycol (EG), bovine serum albumin (BSA), glucose, lysine, tyrosine, potassium ferricyanide (K_3_[Fe(CN)_6_]) and potassium ferrocyanide (K_4_[Fe(CN)_6_]), MAX phase TI_3_ALC_2_, hydrochloric acid (HCL) and lithium fluoride (LiF) were purchase from Sigma Chemical Co (St. Louis, MO). DI water was purified by a Milli-Q system (Deionized water, MΩ•cm @ 25 °C). The photoresist AZ9260 was purchased from MicroChem (USA). The phosphate buffered saline (PBS), (3-Glycidyloxypropyl) trimethoxysilane (GOPS) and other reagents were provided by Aladdin Chemicals Co., Ltd.

### Synthesis of MXene

The Ti_3_C_2_ nanosheets was progressively synthesized by etching Al from Ti_3_AlC_2_ in solution of hydrochloric acid according to reported literatures [25, 37, 38]. Pristine MAX phase was milled for 8 h with the weight ratio of 10: 1 (speed of 450 RPM), and then the TI_3_AlC_2_ was filtered by sieve. Typically, 2 g of LiF was added in 50 mL of HCl under and stirred magnetically for 15 min. Then 3 g of processed Ti_3_AlC_2_ powered slowly added into the mixture and heated at 45 °C for 10 h with stirring. The obtained solution was rinsed continuously with DI water until of pH of ~ 6.5. Subsequently, the resulting supernatant was collected and stored at 4 °C for the subsequent usage.

### Preparations of MXene@PEDOT: PSS nanocomposites

To realize the transistor channels, we prepared a mixture of PEDOT: PSS with d-sorbitol (40% by volume), 1% GOPS (by volume, as a crosslinker), 0.1% DBSA (by volume, for the improvement of film processing and wettability) and DMSO (5% by volume). Then, the colloidal MXene was added into the mixture of PEDOT: PSS. Finally, nanoporous polypropylene membranes (Millipore Corp, USA) was used to filter the MXene@PEDOT: PSS nanocomposites at 4 °C for the subsequent usage.

### Fabrication of isMOECTs biosensor

The fabrication of biosensor included the ultraviolet lithography, deposition of metal (thermal evaporation technique) and spin-coating of MXene@PEDOT: PSS nanocomposites. Before the ultraviolet lithography, the silicon substrate was rinsed with isopropanol and water. Then the substrates were properly dried and exposed O_2_ plasma for 3 min. Subsequently, a customized shadow mask with interdigitated multiple spirals was prepared. After that, the substrate was photolithographically patterned by photoresist AZ9260 (MicroChem) with a positive tone lift-off process. For the deposition of metal layer, the metal interconnection Cr/Au source, drain electrodes (thicknesses: 6 nm/80 nm) were thermally evaporated evaporation on silicon substrates. Consequently, the mixture of MXene@PEDOT: PSS nanocomposites was spin-coated and anneal at 150 ℃ in nitrogen environment for 2 h. Finally, the devices were rinsed with ID water for three times, and purged with nitrogen gun before use and stored at 4 °C.

### Apparatus

The morphological scanning electroni microscopy (SEM) characterization was conducted by S-4800 (Hitachi) by 10000x (voltage of 12 kV). The Data physics SZ-CAMA1was used to analyze the contact angle, with 20 µL of DI water added on the isMOECTs biosensor for observations. The morphological characterization of surface isMOECTs biosensors was conducted by Bruker Dimension EdgeTM with frequency range of 100–400 kHz. The transfer characteristics of biosensor isMOECTs biosensor was measured by Keithley 2510 (Tektronix Co Ltd).

The electrochemical analysis were carried out on an electrochemical workstation (CHI660e, CH Instrument, Shanghai). The scanning voltage was defined from 0 to 450 mV, 20 mV pulse amplitude of, 30 ms pulse width to perform the DPV analysis. The EIS analysis was performed with the parameters of 0.1–20,000 frequency and 10 mV pulse amplitude. For the parameters and settings of isMOECTs measurements, the metal interconnection electrodes of the OECTs were connected to sourcemeter instrument (Keithley 2510, and a MATLAB program was designed to control voltages and record the changes of data.

To characterize the responses of isMOECTs various concentrations of fPSA/tPSA protein, transfer characteristics of isMOECTs was selected to under following parameters: a fixed V_DS_ of 0.08 V under gate voltages ranging from 0–1.4 V. The output characteristics were performed under fixed at V_g_ of 0.9 V at various drain voltages (V_D_: 0–0.2 V).

### Harvesting of biosamples and statistical analysis

The Pudong New Area People’s Hospital affiliated to Shanghai University of Medicine and Health Sciences approved all clinical procedures in this project (No: PRYLZ 2018–039-A). For the procedures of biosamples harvesting, biosamples (5 mL) were centrifuged for 10 min with parameter of 1500 × *g* and stored at -80 ℃ for further determination. The receiver operating characteristics curve was analyzed by Graphpad Prism (V9.1.2.226).

## Results and discussion

### Preparation of MXene@PEDOT: PSS and design of isMOECTs

**Fig.1:**
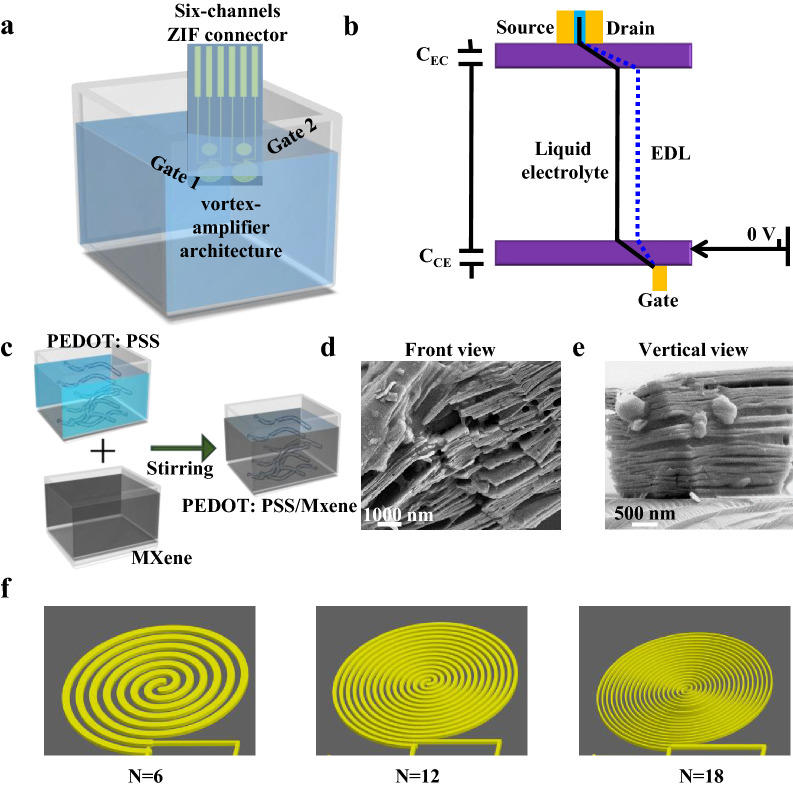
Illustrative schematic of isMOECTs biosensors for quantification of fPSA/tPSA. **a** Typical configurations of isMOECTs biosensor, and **b** corresponding equivalent circuit. **c** The preparation procedures for MXene@PEDOT: PSS nanocomposites. SEM characterizations of MXene@PEDOT: PSS via **d** front view and **e** vertical view. **f** The configurations of isMOECTs biosensors with different number of interdigitated spirals

We performed the synthesis procedures of MXene@PEDOT: PSS semiconductor membrane and the preparation of isMOECTs biosensor in Fig. 1. The Fig. 1a illustrated the key components of isMOECTs biosensor, including six-channel ZIF connector, dual organic electrochemical transistors with respective interdigitated spiral-amplifier architecture. Figure 1b was the equivalent circuit between the channel of isMOECTs and the gate electrode. For the synthesis of MXene@PEDOT: PSS, the crucial element is the synthesis of micropatterned MXene with intercalation and delamination of layered architecture and subsequent structural rearrangement with PEDOT: PSS, which is significant for amplification of amperometric signal of isMOECTs biosensor. As displayed in Fig. 1c, the MXene@PEDOT: PSS semiconducting materials were structurally combined under vigorous stirring, while the PEDOT: PSS recombined with the intercalation/delamination layer of MXene. We conducted the scanning electron microscopy (SEM) of MXene@ PEDOT: PSS by the front view (Fig. 1d) and vertical view (Fig. 1e) to validate the morphological and structural arrangement of MXene @PEDOT: PSS and pure MXene (Additional file 1: Fig. S1). Another key characteristic of isMOECTs biosensor for ultrasensitive determination of fPSA/tPSA is interdigitated multiple spirals, which displayed in Fig. 1f with different number of spirals for amperometric-signal amplification.

### Functionalization of isMOECTs

**Fig. 2:**
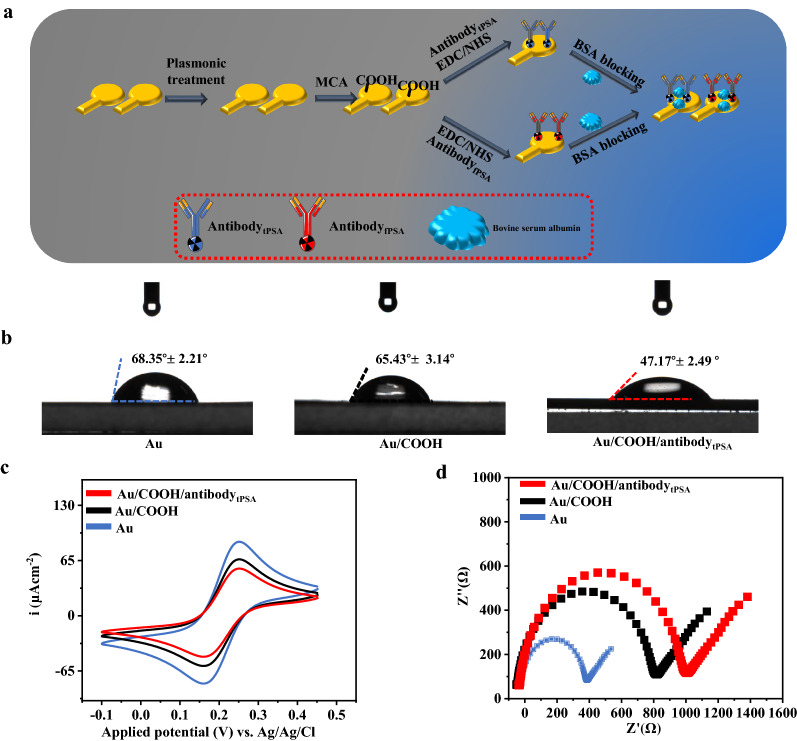
Representations of layer-by-layer functionalizations and characterizations of gate electrode on isMOECTs biosensor. **a** The modification procedures, **b** EIS, **c** CV and **d** contact angle analysis for the gate electrode.

For the specificity of isMOECTs biosensor, we performed fPSA/tPSA functionalizations on the dual gate electrodes in Fig. 2a. We performed the contact angles, cyclic voltammetry (CV), electrochemical impedance and atomic force microscopy (AFM) to characterize the modification processes. For the contact angle (Fig. 2b), the value decreased from 68.35° (blue, Fig. 2b) to 65.43° (black, Fig. 2b) and 47.17° (red, Fig. 2b), respectively, after modification of MCA and anti-tPSA, due to the high hydrophilicity of MCA and anti-tPSA. For the cyclic voltammetry, the peak current declined from 85.31 μA (blue curve in Fig. 2c) to 66.12 μA (black curve in Fig. 2c) and 55.41 μA (red curve in Fig. 2c), respectively. We also conducted the investigation of scan rate-dependency study (Additional file 1: Fig. S2), revealing a linear behavior (I_redox_ = 31.171 × V^1/2^—18.031, R^2^ = 0.9947) between the electrochemical current and the scan rate (V). For the EIS (Fig. 2d), the R_et_ demonstrated 385.4 Ω (blue curve), 812.3 Ω (black curve) and 1038.4Ω (red curve) for Au, Au/COOH, Au/COOH/anti-tPSA. The trend of CV and EIS analysis could be attributed to the blocking of electron via MCA and anti-tPSA. We also conducted the AFM to observe the morphological changes in Additional file 1: Fig. S3. The calculated root mean square value reflects the morphological characteristics of isMOECTs biosensor, including the bare gate electrode (7.8 ± 1.9 nm), Au/MCA (46.3 ± 8.5 nm), Au/MCA/anti-tPSA (135.3 ± 19.5 nm), which validated functionalization procedures. Abovementioned characterizations (contact angle, CV, EIS and AFM) demonstrated successful modification of isMOECTs biosensor for the specific quantifications of fPSA/tPSA.

### Optimizations of isMOECTs and its detection performance

**Fig.3:**
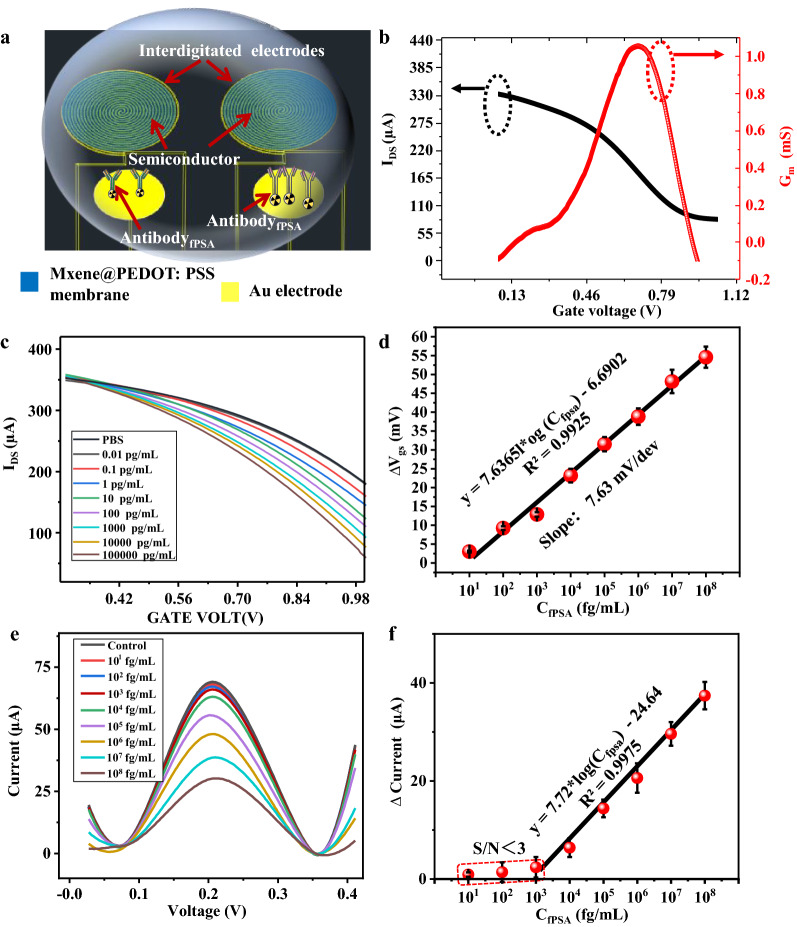
Depiction of quantification mechanism and performance. **a** The conceptual mechanism of isMOECTs for the determination of fPSA/tPSA and **b** typical transfer characteristics with transconductance. **c** The transfer characteristics response of the isMOECTs biosensor to the addition of fPSA, **d** drain current as a function of fPSA. **e** Traditional DPV measurement of the Au gate electrode as the working electrode in the incubation of a series of fPSA. **f** The calibration plot of the DPV testing as a function of fPSA. Each of error in panels d and f is three replicated analysis (n = 3)

To achieve ultrasensitive determination performance of isMOECTs biosensor, we optimized the ratio of MXene/PEDOT: PSS and the architecture of interdigitated multiple spirals. As show in Additional file 1: Fig. S4, we performed the ratio of in 2:1, 1:1, 1.5:1 and 2:1, and the ratio of 1:1 demonstrated highest transconductance in the characterization of transfer curve. For the optimization of spiral architecture, we selected the number of 6, 12, 18, and the number of 12 exhibited highest amperometric amplification of isMOECTs biosensor with the maximum transconductance of 0.99 mS (Additional file 1: Figs. S5 and S6). Considering the bioaffinity between the target molecules (fPSA and tPSA) and isMOECTs biosensor, we also optimized parameters among the concentration of antibody (20 μg/mL), pH value of 7.0, temperature of 37 ℃ and incubation of 40 min for optimum configurations of isMOECTs (Additional file 1: Fig. S7). Based on above optimizations. We performed typical quantifications of fPSA/tPSA as Fig. 3a with corresponding transfer characteristics and transconductance in Fig. 3b. Subsequently, as shown in Fig. 3c, d, we recorded the sensitive determination of fPSA with wide linear wide of 10 fg/mL-100 ng/mL (the detection limit of 5.33 fg/mL), and the corresponding linear regression curve was △V_gs_ = 7.63 × log (C_fPSA_)-6.69, R^2^ = 0.992 with the slope of 8.89 mV/dev. The detection limit of biosensor was calculated at a signal/noise ratio of 3 according to previous literatures [39, 40]. For the purpose of comparisons, the typical gate electrode with anti-fPSA functionalization was to detect fPSA via differential pulse voltammetry. As demonstrated in Fig. 3e, f, the determination performance of DPV technique achieved the detection range of 1000 fg/Ml–100 ng/mL, with the detection limit of 432.5 fg/mL. The tPSA quantification performance of isMOECTs and its corresponding DPV results were also illustrated in Additional file 1: Fig. S8. Our purposed isMOECTs demonstrated higher sensitivity than conventional electrochemical methods and other type biosensors (such as electrochemical impedimetric immunosensors, bead-based fluorescence assay and reflectance spectroscopy) with summarized comparisons in Additional file 1: Table S1, which could be attributed to the combination of micropatterned MXene@PEDOT: PSS and interdigitated multiple spiral architecture.

### Selectivity and reproducibility

**Fig.4:**
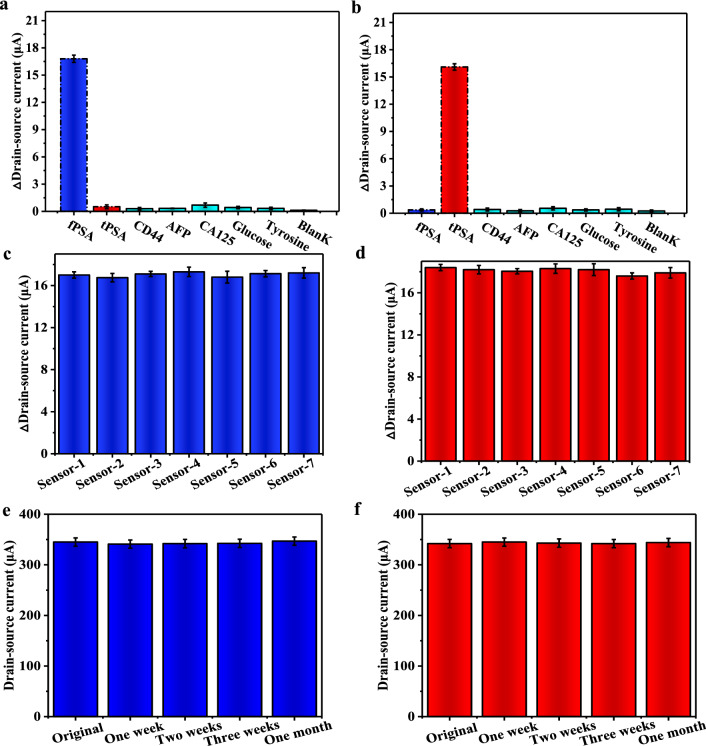
Performance evaluations. **a** The selectivity, **c** reproducibility and **e** stability assessment of isMOECTs biosensors for quantification of fPSA. **b** The selectivity, **d** reproducibility and **e** stability assessment of isMOECTs biosensors for determination of tPSA. Each of error in panels a-f is three replicated analysis (n = 3)

We selected three high-weight molecules and two small-molecules as interferences to evaluate the selectivity of isMOECTs biosensor. As illustrated in Fig. 4a, the anti-fPSA-functionalized isMOECTs biosensor displayed high specificity for quantification of 10 ng/mL fPSA with the value of 16.8 ± 0.43 μA, which was obviously high than 10 ng/mL tPSA (0.51 ± 0.21 μA), 100 ng/mL CD44 (0.31 ± 0.11 μA), 100 ng/mL AFP (0.33 ± 0.15 μA), 100 ng/mL CA125 (0.69 ± 0.24 μA), 100 ng/mL glucose (0.42 ± 0.13 μA), 100 ng/mL tyrosine (0.33 ± 0.12 μA) and blank (0.11 ± 0.04 μA), demonstrating high specificity of isMOECTs biosensor for quantification of fPSA. Moreover, anti-fPSA-functionalized isMOECTs biosensor displayed high specificity for quantification of tPSA against other interferences (Fig. 4b).

For the reproducibility, we recorded amperometric signals of seven anti-fPSA-functionalized isMOECTs biosensors and seven anti-tPSA-functionalized isMOECTs biosensors with RSD values of 1.19 and 1.51%, respectively, demonstrating acceptable reproducibility of proposed isMOECTs biosensors (Fig. 4c, d). For the stability, compared with original status, the amperometric signal exhibited negligible variation with decline by < 5% (Fig. 4e, f), affording applicable stability of proposed isMOECTs biosensor.

### Clinical performance

**Fig.5:**
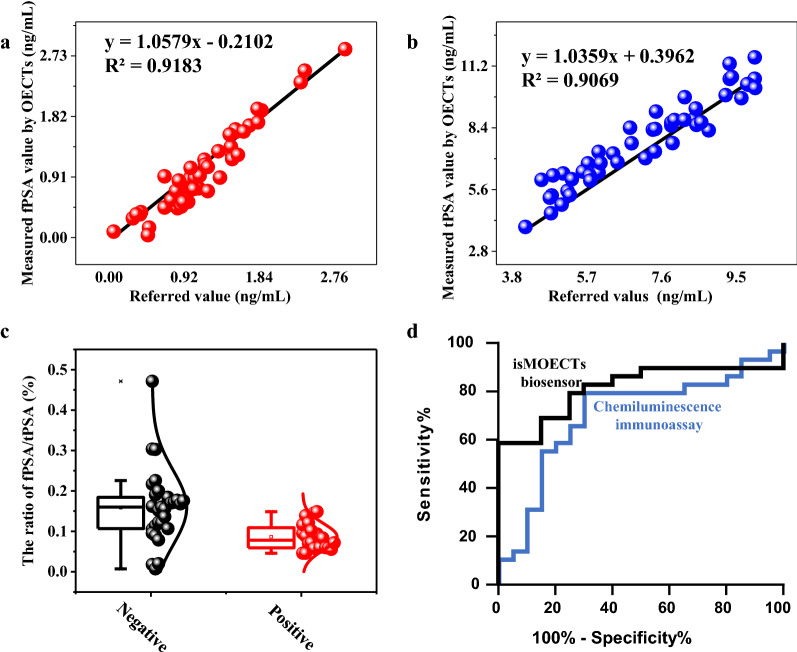
Clinics assessments for 49 patient biosamples. **a** Correlation analysis between reference values (Beckham DXI600) and measured value (via isMOECTs) for quantification of **a** fPSA and **b** tPSA. **c** The T-test discrimination for the ratio of fPSA/tPSA among all biosamples. **d** The comparisons of receiver operating characteristic between the chemiluminescence immunoassay (blue curve, Beckham DXI600) and the isMOECTs biosensors (black curve)

In clinics, the isMOECTs biosensors were subsequently applied in a cohort of 49 human serum (Additional file 1: Table S2), and the biosamples were standardly harvested according to typical protocol (in Experiment section). As displayed in Fig. 5a, b, and isMOECTs biosensor achieved high correlation with reference value for the quantification of fPSA and tPSA, respectively. Moreover, we calculated all ratio value of fPSA/tPSA in Fig. 5c with p < 0.01. Finally, we performed the receiver operating characteristic (ROC) analysis in Fig. 5d. The isMOECTs biosensors afforded good sensitivity (75.8%) and specificity (75.0%), as well as excellent area under the curve (AUC) value of 0.8138 (confidence interval: 95%) against referenced clinical chemiluminescence immunoassay (Beckham DXI600), confirming the potential applications of isMOECTs in clinics.

## Conclusions

In summary, isMOECTs biosensor was used in this work as a potential diagnostic tool for sensitive and specific quantification of fPSA/tPSA in prostate cancer patients. By combination of the micropatterned MXene@PEDOT: PSS and multiple spiral architecture, the isMOECTs biosensor afforded improved assay detection sensitivity as well as high specificity, reproducibility and reliability. Moreover, the isMOECTs displayed excellent area under the curve (AUC) value of 0.8138, confirming the potential applications of isMOECTs in clinics. This is first demonstration of MXene-based spiral-amplifier organic electrochemical transistor biosensor for human (prostate) cancer diagnosis and our strategy would be extended to detect other biomarkers in various types of cancer or for liquid biopsy, paving a convenient and versatile platform for quantification of cancer-relevant biomarkers.

## Supplementary Information

**Additional file 1: Table S1.** Comparisons of performance. **Table S2**. Clinical information of cohort. **Fig. S1.** The SEM characterization of pristine MXene. **Fig. S2.** CV analysis of gate electrode. **Fig. S3.** The AFM analysis of electrodes. **Fig. S4.** The optimizations of ratio of MXene/PEDOT: PSS nanocomposites. Fig. S5. The optimizations of number of spiral. **Fig. S6.** The configurations and optical image of isMOECTs. **Fig. S7.** The optimization of parameters for fabrication of biosensor. **Fig. S8.** The analysis and corresponding calibration of tPSA.

## Data Availability

All data generated or analyzed during this study are included in this article.
