# Supplementary material for: MXene-assisted organic electrochemical transistor biosensor with multiple spiral interdigitated electrodes for sensitive quantification of fPSA/tPSA
Source: J Nanobiotechnology. 2021 Nov 24;19:386. doi: 10.1186/s12951-021-01121-x (PMC8611845; doi:10.1186/s12951-021-01121-x)
Supplement: Supplementary file 1 — Additional file 1: Table S1. Comparisons of performance. Table S2. Clinical information of cohort. Fig. S1. The SEM characterization of pristine MXene. Fig. S2. CV analysis of gate electrode. Fig. S3. The AFM analysis of electrodes. Fig. S4. The optimizations of ratio of MXene/PEDOT: PSS nanocomposites. Fig. S5. The optimizations of number of spiral. Fig. S6. The configurations and optical image of isMOECTs. Fig. S7. The optimization of parameters for fabrication of biosensor. Fig. S8. The analysis and corresponding calibration of tPSA. [file 12951_2021_1121_MOESM1_ESM.pdf]

MXene-assisted organic electrochemical transistors biosensor with multiple spiral interdigitated electrodes for sensitive determination of fPSA/tPSA

Yi-Cheng Zhu[a], Biao Cai[b], Quan Jiang[a], Yuan Zhang[a], Jianjun Sha[b, *], Shaowei Xie[c, *]

[a]Department of Ultrasound, Pudong New Area People's Hospital affiliated to Shanghai University of Medicine and Health Sciences, Shanghai 201200, China;

[b]Department of Urology, Renji Hospital, Shanghai Jiao Tong University School of Medicine, Shanghai 200127, China;

[c]Department of Ultrasound, Renji Hospital, Shanghai Jiao Tong University School of Medicine, Shanghai 200127, China.

***Corresponding author:** Prof Shaowei Xie, Tel: 0086-21-68383396; E-mail: xieshaowei@renji.com. Prof Jianjun Sha, Tel: 0086-21-68383396; E-mail: shajianjunuro@163.com.

This Supplementary Information includes:

S1 to S8

Tables S1 to S2

References

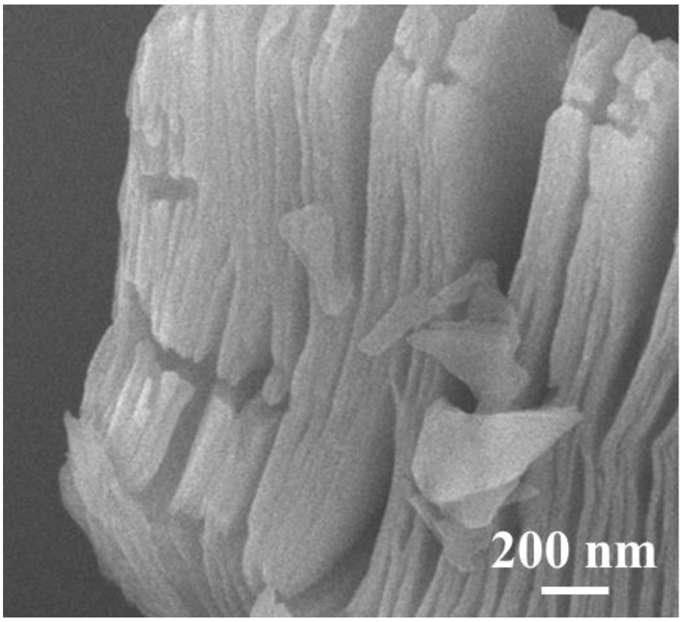

S1. The SEM characterization of pristine MXene.

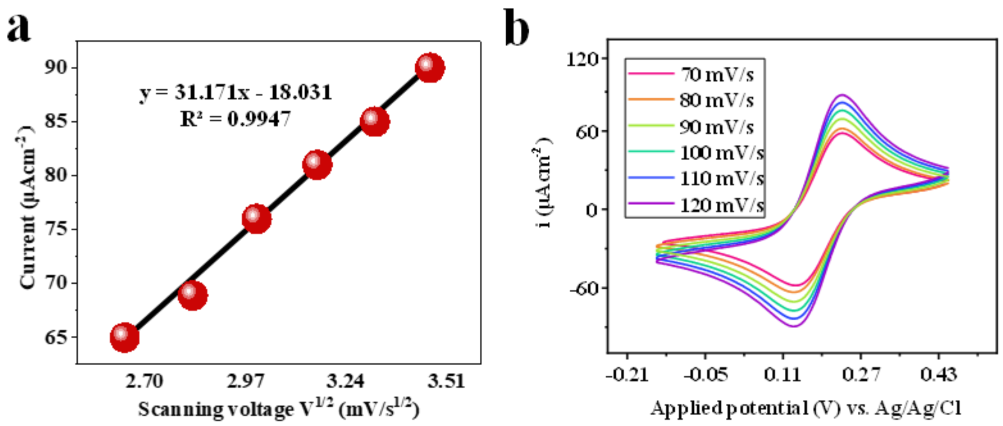

S2. a) Cyclic voltammetry of gate electrode under various scan rate and b) corresponding relationship between the scan rates and the current. The CV analysis were conducted in a mixture solution of 5 mM potassium ferricyanide/potassium ferrocyanide ($K_3[Fe(CN)_6]$)/$K_4[Fe(CN)_6]$).

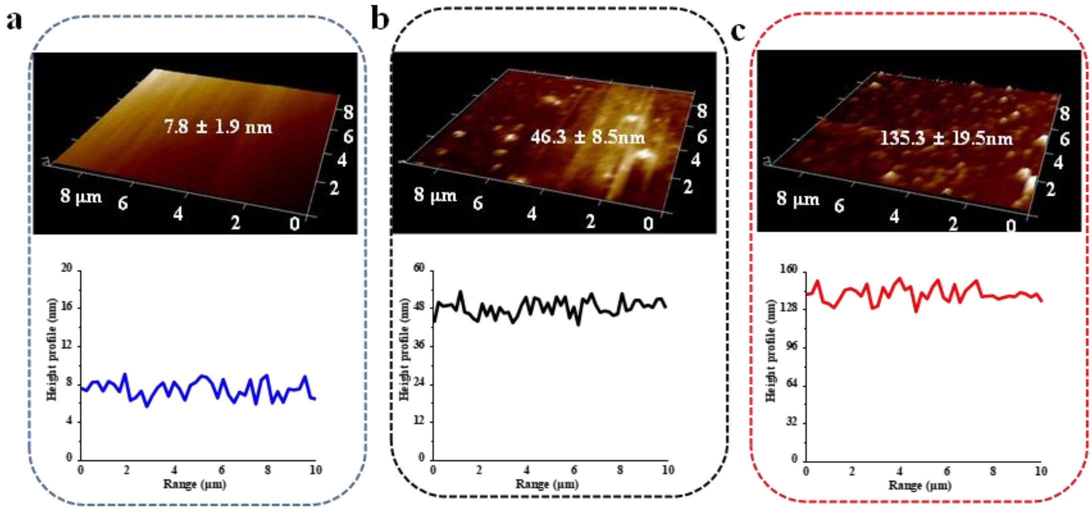

S3. The AFM analysis of a) Au, b)Au/COOH, and c) Au/COOH/antibody$_{tPSA}$.

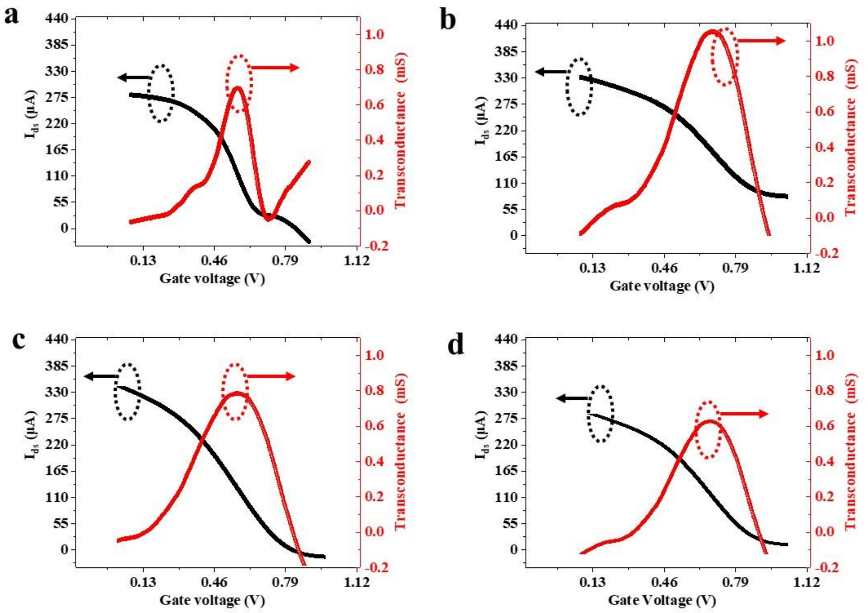

S4. The optimizations of ratio of MXene/PEDOT: PSS nanocomposites. The transfer characteristics of isMOECTs and corresponding transconductance with the ratio of a) 2:1, b) 1:1, c) 1:1.5 and d) 1:2.

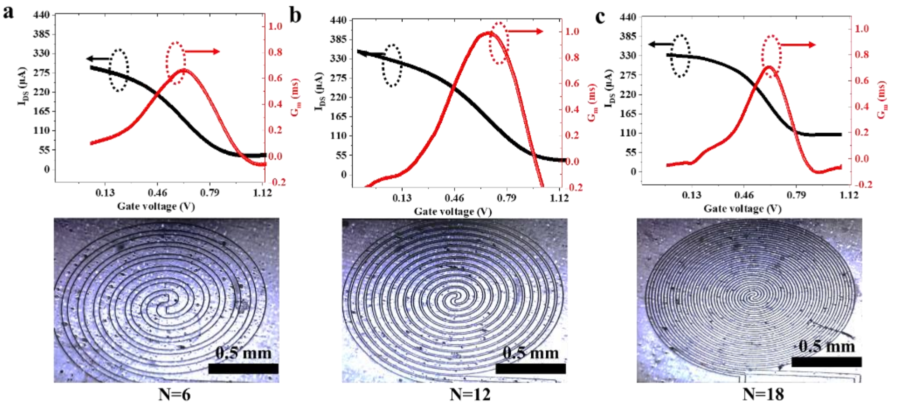

S5. The optimizations of number of spiral for isMOECTs biosensors. The transfer characteristics of isMOECTs and corresponding transconductance with the number of spiral of a) N=6, b) N=12, c) N=18.

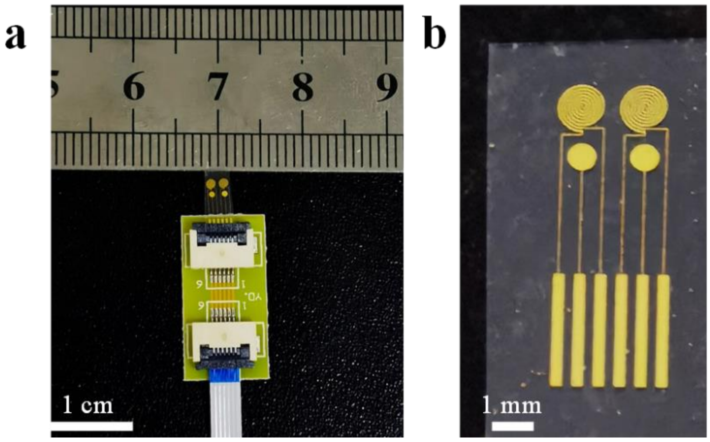

S6. a) The configurations and b) optical image of isMOECTs.

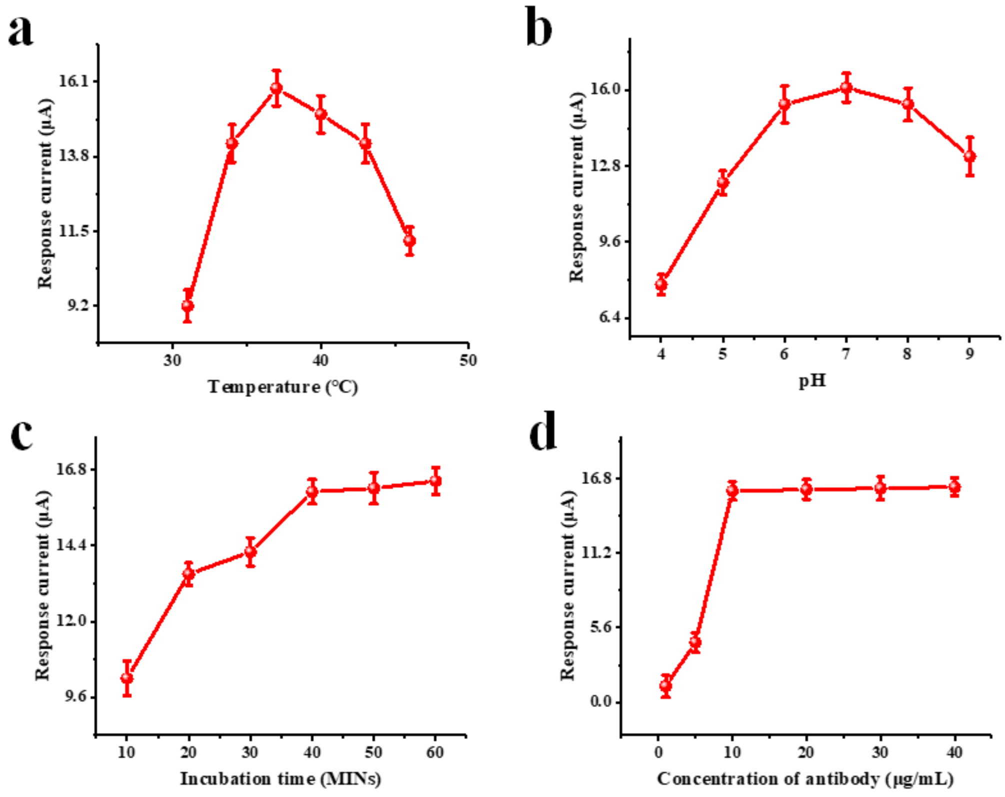

S7. The optimizations of a) temperature, b) pH value, c) incubation time and d) concentration of antibody for the isMOECTs biosensors.

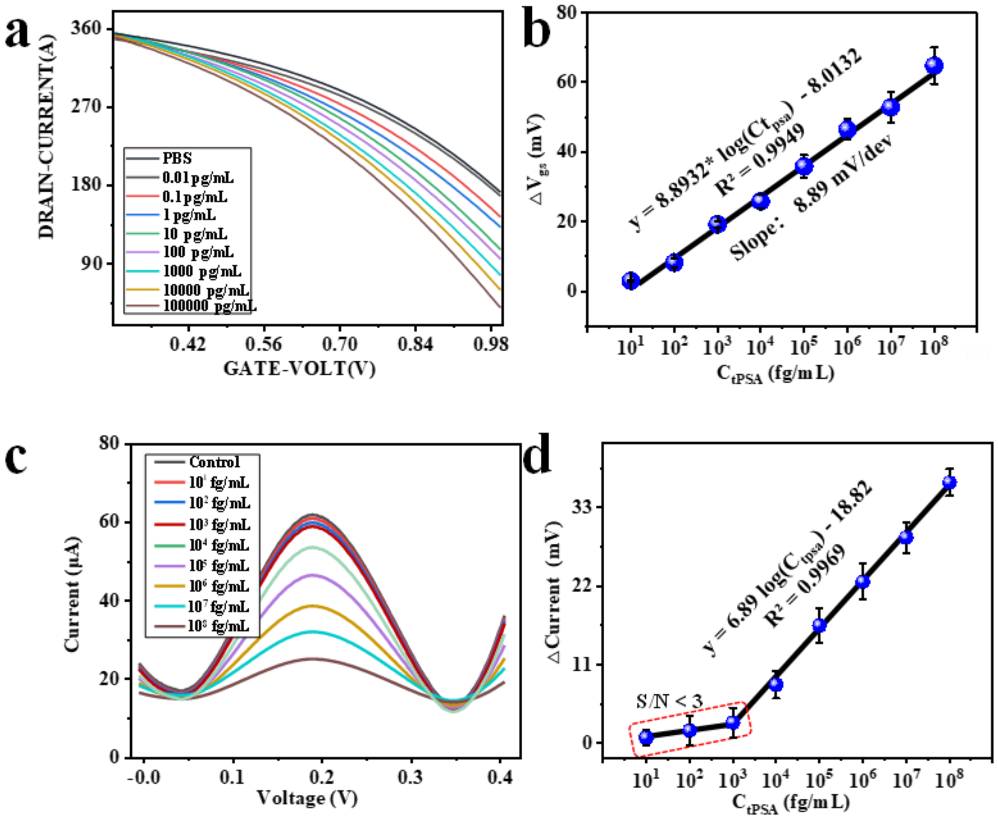

S8. a) The transfer characteristics responses of the isMOECTs biosensors to the addition of tPSA, b) drain current as a function of tPSA. c) Traditional DPV measurement of the Au gate electrode as the working electrode in the incubation of a series of tPSA. d) The calibration plot of the DPV testing as a function of tPSA. Each of error in panels b and d is three replicated analysis (n=3).

Table S1. Comparisons of performance.

Technique	Detection limit	Detection range	Diagnosis analysis	Source
Eectrochemical impedimetric immunosensors	3.0 fg/mL for fPSA; 4.0 fg/mL for tPSA;	0.02 pg/mL-200 ng/mL for fPSA: 0.02 pg/mL-200 ng/mL for tPSA;	-	(Han et al., 2019)
Bead-based fluorescence assay	3.8 pg/mL for fPSA; 22.5 pg/mL for tPSA;	10-1000 pg/mL for fPSA; 10-10000 pg/mL for tPSA;	-	(Cao et al., 2018)
Reflectance spectroscopy	0.2 ng/mL for tPSA	0.5-20 ng/ml for tPSA; 0.5-20 ng/mL for fPSA;	-	(Koukouvinos et al., 2015)
isMOECTs	5.3 fg/mL for fPSA; 4.8 fg/mL for tPSA;	0.01 pg/mL-100 ng/mL for fPSA: 0.01 pg/mL-100 ng/mL for tPSA;	AUC of with 0.8138 with sensitivity of 75.8% and specificity of 75.0%;	This work

Table S2. Clinical information of cohort.

Number	Age	tPSA[1]	fPSA[1]	f/t ratio	tPSA by isMOECTs	fPSA by isMOECTs	f/t ratio by isMOECTs	Cancer
**1**	63	6.37	0.88	13.74%	7.23	0.69	9.51%	PCa[2]
**2**	67	9.97	1.51	15.15%	10.62	1.23	11.62%	PCa[2]
**3**	73	9.34	1.01	10.82%	10.62	0.94	8.82%	PCa[2]
**4**	69	9.39	0.74	7.88%	10.70	0.49	4.59%	PCa[2]
**5**	74	4.81	1.10	22.87%	5.33	0.74	13.87%	PCa[2]
**6**	64	5.10	0.84	16.47%	6.33	0.44	6.97%	PCa[2]
**7**	83	9.33	1.49	16.01%	11.30	1.37	12.08%	PCa[2]
**8**	70	4.84	0.68	14.05%	6.24	0.45	7.24%	PCa[2]
**9**	81	4.55	0.38	8.35%	6.04	0.35	5.79%	PCa[2]
**10**	70	7.85	0.89	11.34%	8.50	0.79	9.34%	PCa[2]
**11**	61	9.97	1.21	12.14%	11.59	0.70	6.03%	PCa[2]
**12**	70	7.85	0.99	12.61%	8.64	0.88	10.20%	PCa[2]
**13**	79	5.73	0.76	13.26%	6.79	0.57	8.35%	PCa[2]
**14**	70	6.00	1.20	20.00%	7.31	1.06	14.55%	PCa[2]
**15**	69	5.32	1.36	25.56%	6.07	0.90	14.87%	PCa[2]
**16**	80	5.60	0.39	7.00%	6.41	0.38	5.96%	PCa[2]
**17**	85	5.84	0.29	5.00%	6.33	0.29	4.58%	PCa[2]
**18**	65	7.46	0.97	13.00%	9.13	0.54	5.87%	PCa[2]
**19**	53	6.80	0.88	13.00%	8.40	0.47	5.53%	PCa[2]
**20**	73	8.19	0.82	10.01%	9.79	0.70	7.18%	PCa[2]
**21**	68	5.21	1.05	20.15%	5.54	0.90	16.19%	BPH[3]
**22**	68	7.43	1.55	20.86%	7.32	1.59	21.67%	BPH[3]
**23**	62	8.40	0.68	8.10%	8.76	0.92	10.50%	BPH[3]
**24**	63	5.06	0.95	18.77%	4.92	0.56	11.37%	BPH[3]
**25**	64	8.49	1.55	18.26%	8.53	1.63	19.05%	BPH[3]
**26**	64	7.88	1.17	14.85%	7.70	1.17	15.19%	BPH[3]
**27**	61	9.63	1.11	11.53%	9.75	0.93	9.54%	BPH[3]
**28**	60	5.27	0.86	16.22%	5.35	0.86	16.00%	BPH[3]
**29**	71	7.38	1.34	18.16%	8.31	1.30	15.60%	BPH[3]
**30**	70	9.82	2.35	23.93%	10.34	2.33	22.56%	BPH[3]
**31**	70	9.78	1.87	19.12%	10.38	1.91	18.41%	BPH[3]
**32**	63	9.23	1.72	18.63%	9.88	1.68	17.03%	BPH[3]
**33**	68	4.14	0.34	8.09%	3.91	0.35	8.96%	BPH[3]
**34**	73	8.47	1.65	19.48%	9.27	1.59	17.18%	BPH[3]

**35**	58	6.00	1.82	30.33%	6.37	1.93	30.35%	BPH[3]
**36**	61	7.93	1.48	18.66%	8.75	1.55	17.70%	BPH[3]
**37**	78	7.19	1.51	21.00%	7.02	1.18	16.80%	BPH[3]
**38**	72	9.98	1.58	15.83%	10.21	1.24	12.18%	BPH[3]
**39**	66	5.77	1.18	20.45%	6.24	1.10	17.57%	BPH[3]
**40**	60	7.44	0.49	6.59%	8.34	0.15	1.80%	BPH[3]
**41**	64	8.80	2.40	27.27%	8.29	2.51	30.27%	BPH[3]
**42**	67	8.18	1.21	14.79%	8.76	1.06	12.13%	BPH[3]
**43**	80	8.61	1.83	21.21%	8.65	1.73	20.02%	BPH[3]
**44**	68	6.05	0.91	15.01%	6.80	0.54	7.88%	BPH[3]
**45**	71	6.84	1.00	14.62%	7.70	1.05	13.60%	BPH[3]
**46**	46	5.79	2.90	50.00%	6.01	2.83	47.13%	BPH[3]
**47**	62	6.48	1.04	16.00%	6.84	0.73	10.66%	BPH[3]
**48**	57	4.76	0.48	10.00%	5.23	0.04	0.72%	BPH[3]
**49**	57	4.79	0.06	1.19%	4.53	0.09	1.99%	BPH[3]

[1] The tPSA and fPSA were measured by chemiluminescence immunoassay (Beckham DXI600);

[2] PCa is the prostate cancer;

[3] BPH is the benign prostatic hyperplasia as the control.

Reference

Cao, _D_, Li, C.Y., Qi, C.B., Chen, H.L., Pang, D.W., Tang, H.W., 2018. Multiple optical trapping assisted bead-array based fluorescence assay of free and total prostate-specific antigen in serum. Sensors Actuators B Chem. 269, 143-150.

Han, L., Wang, D., Yan, L., Petrenko, V.A., Liu, A., 2019. Specific phages-based electrochemical impedimetric immunosensors for label-free and ultrasensitive detection of dual prostate-specific antigens. Sensors Actuators, B Chem. 297. https://doi.org/10.1016/j.snb.2019.126727

Koukouvinos, G., Petrou, P.S., Misiakos, K., Drygiannakis, D., Raptis, I., Goustouridis, D., Kakabakos, S.E., 2015. A label-free flow-through immunosensor for determination of total- and free-PSA in human serum samples based on white-light reflectance spectroscopy. Sensors Actuators B Chem. 209, 1041-1048.
